# Dynamic and Static Exercises Differentially Affect Plasma Cytokine Content in Elite Endurance- and Strength-Trained Athletes and Untrained Volunteers

**DOI:** 10.3389/fphys.2017.00035

**Published:** 2017-01-30

**Authors:** Leonid V. Kapilevich, Anna N. Zakharova, Anastasia V. Kabachkova, Tatyana A. Kironenko, Sergei N. Orlov

**Affiliations:** ^1^Department of Sports Tourism Sports Physiology and Medicine, National Research Tomsk State UniversityTomsk, Russia; ^2^Department of Sports Disciplines, National Research Tomsk Polytechnic UniversityTomsk, Russia; ^3^Laboratory of Physical Chemistry of Biomembranes, Faculty of Biology, M. V. Lomonosov Moscow State UniversityMoscow, Russia

**Keywords:** interleukins, leukemia inhibitory factor, dynamic exercise, static exercise, trained athletes and untrained volunteers

## Abstract

Extensive exercise increases the plasma content of IL-6, IL-8, IL-15, leukemia inhibitory factor (LIF), and several other cytokines via their augmented transcription in skeletal muscle cells. However, the relative impact of aerobic and resistant training interventions on cytokine production remains poorly defined. In this study, we compared effects of dynamic and static load on cytokine plasma content in elite strength- and endurance-trained athletes vs. healthy untrained volunteers. The plasma cytokine content was measured before, immediately after, and 30 min post-exercise using enzyme-linked immunosorbent assay. Pedaling on a bicycle ergometer increased IL-6 and IL-8 content in the plasma of trained athletes by about 4- and 2-fold, respectively. In contrast to dynamic load, weightlifting had negligible impact on these parameters in strength exercise-trained athletes. Unlike IL-6 and IL-8, dynamic exercise had no impact on IL-15 and LIF, whereas static load increases the content of these cytokines by ~50%. Two-fold increment of IL-8 content seen in athletes subjected to dynamic exercise was absent in untrained individuals, whereas the ~50% increase in IL-15 triggered by static load in the plasma of weightlifting athletes was not registered in the control group. Thus, our results show the distinct impact of static and dynamic exercises on cytokine content in the plasma of trained athletes. They also demonstrate that both types of exercises differentially affect cytokine content in plasma of athletes and untrained persons.

## Introduction

Skeletal muscle is a major determinant of postural retention, locomotion, and regulation of energy consumption and production (Frontera and Ochala, 2015). Since the initial reports (Sprenger et al., 1992; Drenth et al., 1995; Nehlsen-Cannarella et al., 1997; Ostrowski et al., 1999), numerous research teams have demonstrated that physical exercise induces elevation of the plasma content of several cytokines, including tumor necrosis factor TNF-α, interleukins IL-1β, IL-6, IL-8, and IL-15, and leukemia inhibitory factor (LIF). The first studies of the muscle endocrine role demonstrated that skeletal muscles are the major source for the exercise-induced increase in plasma IL-6 (Steensberg et al., 2000). It was also reported that IL-6 transcription rate is increased in nuclei isolated from human muscle biopsies after the onset of exercise (Keller et al., 2001). Later, mouse skeletal muscle cell lines, C_2_C_12_ myoblasts, and primary human myotubes subjected to electrical pulse stimulation (EPS) were widely employed as an *in vitro* exercise model for the study of myokine production (Nedachi et al., 2008; Lambernd et al., 2012; Nikolic et al., 2012). Using this approach, it was shown that 24 h exposure of human myotubes to EPS resulted in 200 differentially expressed transcripts, with the highest secretion level of IL-6, IL-8, chemokine (C-X-C motif) ligand 1 (CXCL1), and LIF (Scheler et al., 2013). These findings revealed skeletal muscle as an endocrine organ releasing cytokines and other peptides. They also suggested that these compounds, classified as myokines, have diverse physiological implications (for comprehensive reviews, see Pedersen and Febbraio, 2008, 2012; Iizuka et al., 2014). Indeed, in skeletal muscles, IL-6 acts in autocrine or paracrine manner signaling through IL-6Ra receptors to increase glucose uptake and fat oxidation via phosphorylation of protein kinase B and AMP-activated protein kinase (AMPK), respectively, whereas acting in endocrine manner it provides energy supply via increase in glucose production in the liver and lipolysis in the adipose tissue (Pedersen and Fischer, 2007). IL-15 decreases lipid deposition in preadipocytes and the mass of white adipose tissue (Quinn et al., 2005). LIF induces cell proliferation, which is considered to be essential for proper muscle hypertrophy and regeneration (Broholm and Pedersen, 2010; Srikuea et al., 2011). Considering this, the role of interleukins and other myokines in the beneficial action of exercise in the treatment of metabolic, cardiovascular, lung, and musculoskeletal disorders is widely discussed (Pedersen and Febbraio, 2008; Pedersen and Saltin, 2015).

It should be noted that our knowledge of exercise-induced myokine production is mainly based on data obtained in studies employing cyclic exercise, whereas data on myokine production evoked by static exercise is limited to a few investigations (Karamouzis et al., 2001; Coffey et al., 2006; Louis et al., 2007; Ochi et al., 2011). Considering this, it should be emphasized that cyclic (dynamic) exercise, such as walking, jogging, and swimming, uses large muscle groups representing more than two thirds of the total muscle mass. In contrast to cyclic exercise, weightlifting and static exercises involve smaller muscle groups representing less than one third of the total muscle mass. Joint angle and muscle length do not change during static muscle contractions, but they do change during dynamic muscle contraction. Static muscular load is more tiring for the body and muscles than dynamic muscular load of the same intensity and duration, because static muscular load does not include the phase of relaxation, during which substances can restock resources expended for muscle contraction (Egan and Zierath, 2013).

To the best of our knowledge, there are no reports comparing the action of preliminary training on cytokine production evoked by static and dynamic load. This is important because of substantial individual variability in the magnitude of changes in myokine plasma content after exercise. Thus, in 200 participants of the Western States 160-km Endurance Run, the increment of plasma IL-6 content varied from 5 to 800 pg/ml (Peake et al., 2015). Keeping this in mind, we designed the present study to comparatively analyze the action of dynamic and static load on cytokine plasma content in elite strength- and endurance-trained athletes vs. healthy untrained volunteers.

## Materials and methods

This study employed four groups of young men aged 18–23 years. The weightlifting group (WG) was formed from 10 elite strength-trained athletes. The track and field group (TFG) consisted of 10 elite endurance-trained athletes (middle-distance running). Both control group 1 (CG1) and control group 2 (CG2) included 10 healthy untrained volunteers. The control group was divided into control group 1 and control group 2 randomly.

None of the subjects had acute or chronic pathologies in their anamneses. The athletes of WG and TFG were engaged in sports more than 6 years. All athletes took part in competitions of Russian level and won prizes. The anthropometric data of subjects are presented in Table 1. All participants signed an informed consent to participate in the study and consent to blood sampling. Permission was obtained from the ethical committee of Tomsk State University (registration #11).

**Table 1:** **The general data of athletes and untrained volunteers**.

**Group**	**Age (yr)**	**Stature (cm)**	**Body weight (kg)**	**VOxmax (ml/min/kg)**	**Oxygenation (%)**	**Strength max (leading hand) (kg)**
Weightlifting (WG)	19.9 ± 1.4	177.0 ± 4.5	82.7 ± 10.2	65.4 ± 4.7	96.5 ± 1.2	65.5 ± 5.2
Track and field (TFG)	20.8 ± 1.4	180.2 ± 6.2	73.2 ± 6.9	74.7 ± 5.2	96.3 ± 2.1	58.2 ± 2.3
CG1 − control	19.5 ± 0.7	183.2 ± 5.7	74.5 ± 4.75	52.3 ± 3.3	95.3 ± 1.5	38.1 ± 4.3
CG2 − control	20.2 ± 1.1	179.4 ± 3.1	69.8 ± 3.1	50.0 ± 4.8	97.2 ± 2.6	42.4 ± 5.6

*Means ± S. D. are shown*.

The strength-trained athletes (WG) and the corresponding controls (CG1) carried out static load consisting of once holding a rod at a level below the knees. The rod weight was 50% of the best results shown in the deadlift. The maximum weight was determined in advance, no later than 1 week prior to the study. The procedure for determining the maximum weight was preceded by a warm-up and guidance for working with weights. Instruction was provided by a professional trainer. Before performing the rod-holding procedure, all subjects were well warmed up. The exercises were also carried out under the guidance of the instructor. The rod-holding procedure was stopped at the stage of complete exhaustion and the inability to continue the exercise. The time of rod-holding in the control and weightlifting group was 53.0 ± 15.2 and 61.8 ± 13.5 s, respectively.

Endurance-trained athletes (TFG) and the corresponding control volunteers (CG2) carried out dynamic load by the standard PWC170 test comprised of pedaling at two different power levels. The first stage was pedaling on a bicycle ergometer for 5 min with the power chosen in accordance with the weight of the participants, and it was terminated by a 15-s heart rate measurement. After a 3-min rest, pedaling on the bicycle ergometer was continued for 5 min with the power chosen according to the heart rate measured at the end of the first load. Before the end of the PWC170 test, the 15-s heart rate measurement was repeated. For more details, see Svannshvili et al. (2009). All volunteers were examined in the morning on an empty stomach. A day before the study, the athletes refrained from their training.

To assess the level of exercise intensity the lactate in capillary blood was measured. Measurement of lactate concentration in capillary blood was performed using the portable device Accutrend Plus (Roche Diagnostics, Germany). The indicator of blood lactate is a reliable indicator of the anaerobic metabolism degree during exercise (Allen et al., 2008).

### Blood samples

*Blood samples* were collected before, immediately after, and 30 min after exercise termination using a BD Vacutainer 5-ml vacuum system and 5 ml Vacuette Premium tubes with heparin separation gel (Greiner Bio-One, Austria). Specified intervals for blood collection have been identified in connection with the date that myokines development can increase both directly during exercise (Broholm et al., 2011), and at the certain intervals after physical exercises (Scheler et al., 2013). Within 30 min of blood collection, erythrocytes and white blood cells were sedimented for 10 min at 2000 rpm using an LMC 3000 centrifuge (Biosan, Latvia). The plasma was frozen and stored for no more than 30 days at −20°C.

### Plasma cytokine content

To measure *plasma cytokine content*, we used the high-sensitivity human LIF Platinum ELISA Kit, Human IL-6 Platinum ELISA Kit, and Human IL-8 Platinum ELISA Kit from eBioscience (Austria) and the RayBio® Human IL-15 ELISA Kit (RayBio®, USA) using microwell test strips with flat-bottomed wells (12 × 8 wells) in accordance with the manufacturer's instructions. The microwell strips were incubated on a PST-60HL microplate shaker (Biosan, Latvia) and washed using an Anthos Fluido 2 washer (Biochrom, Great Britain). The sample optical density was measured using an Anthos 2010 spectrophotometer and ADAP+ software (Biochrom, Great Britain) at 450 nm and 620 nm as primary and reference wavelengths, respectively.

### Statistical analysis

All statistical analyses were conducted using SPSS Statistics software (version 17.0; SPSS Inc., Chicago, IL, USA) at a significance level of 0.05. All tests were performed after logarithmic transformation to satisfy the normality assumption. Data are presented as Mean ± Standard Deviation. The results were statistically analyzed using mixed-effect and repeated-measure analysis of variance followed by paired multiple comparisons (mixed ANOVA).

## Results

In all groups, after the static and dynamic load the lactate level was increase in capillary blood (Table 2). In TFG there was an increase of 2.2 times (*p* < 0.001). In weightlifting group after static load the lactate level was increased by 2.1 times (*p* < 0.001). In the control groups after static and dynamic load the lactate level was increased of 2.1 and 2.3 times respectively (*p* < 0.001). In 30 min of exercise, the lactate levels were decreased (*p* < 0.05). However, significant differences between these values and basal levels were preserved (*p* < 0.05). These results demonstrate the same level of physical load in all subjects thus allowing the comparative analysis the differences in cytokine production.

**Table 2:** **The lactate content in capillary blood of athletes and untrained volunteers (mmol/l)**.

	**Blood lactate**	**Weightlifting (WG)**	**Track and field (TFG)**	**CG1 − control**	**CG2 − control**
1.	Baseline level	3.4 ± 0.5	3.9 ± 0.6	3.8 ± 0.4	3.7 ± 0.3
2.	0 min post exercise	7.2 ± 0.9	8.4 ± 0.3	7.9 ± 0.4	8.4 ± 0.5
3.	30 min post exercise	5.0 ± 0.2	6.1 ± 0.4	5.1 ± 0.3	6.3 ± 0.6
P_1, 2_		<0.001	<0.001	<0.001	<0.001
P_1, 3_		<0.05	<0.05	<0.05	<0.05
P_2, 3_		<0.05	<0.05	<0.05	<0.05

*Means ± S. D. are shown*.

### The baseline plasma cytokine content

**Figure 1:**
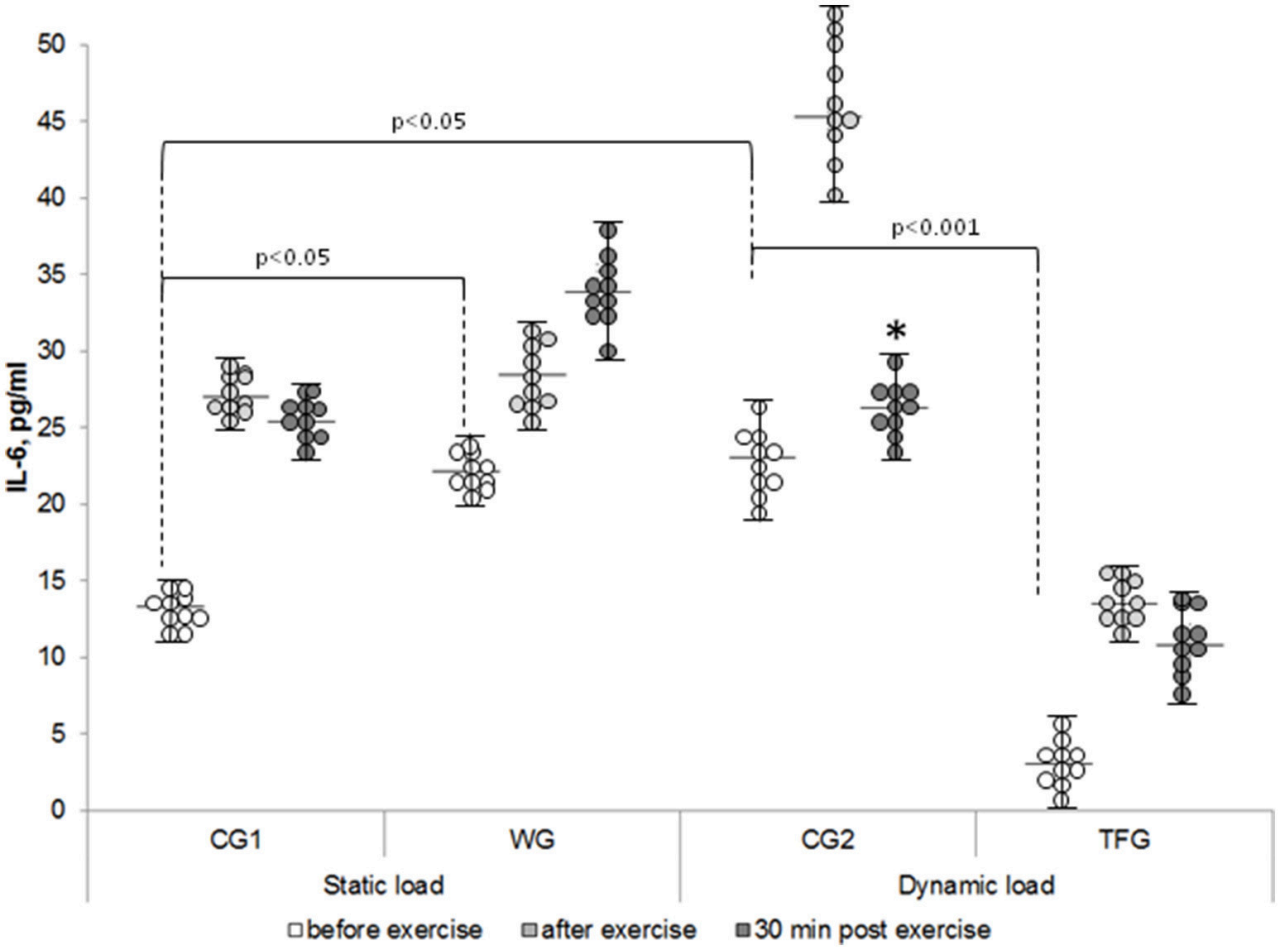
**The individual variability in plasma IL-6 content in the weightlifting (WG), track and field (TFG) athletes and untrained age-matched volunteers**. Horizontal and vertical bars correspond to mean values and standard deviations, respectively. ^*^p < 0.05 compared to the value after exercise.

The lowest baseline IL-6 content of 3 pg/ml was found in the track and field athletes (TFG), which was up to 10-fold less than in the untrained volunteers (CG1 and CG2) and in athletes from the weightlifting group (WG) (*p* < 0.001) (Figure 1). We observed ~1.5-fold elevation of the baseline IL-6 content in CG2 compared to (*p* < 0.05, Figure 1). However, these differences were much less as compared with 5-8-fold decreased baseline IL-6 content seen in the track and field athletes as compared to both control groups and weightlifting athletes (*p* < 0.001, Figure 1).

**Figure 2:**
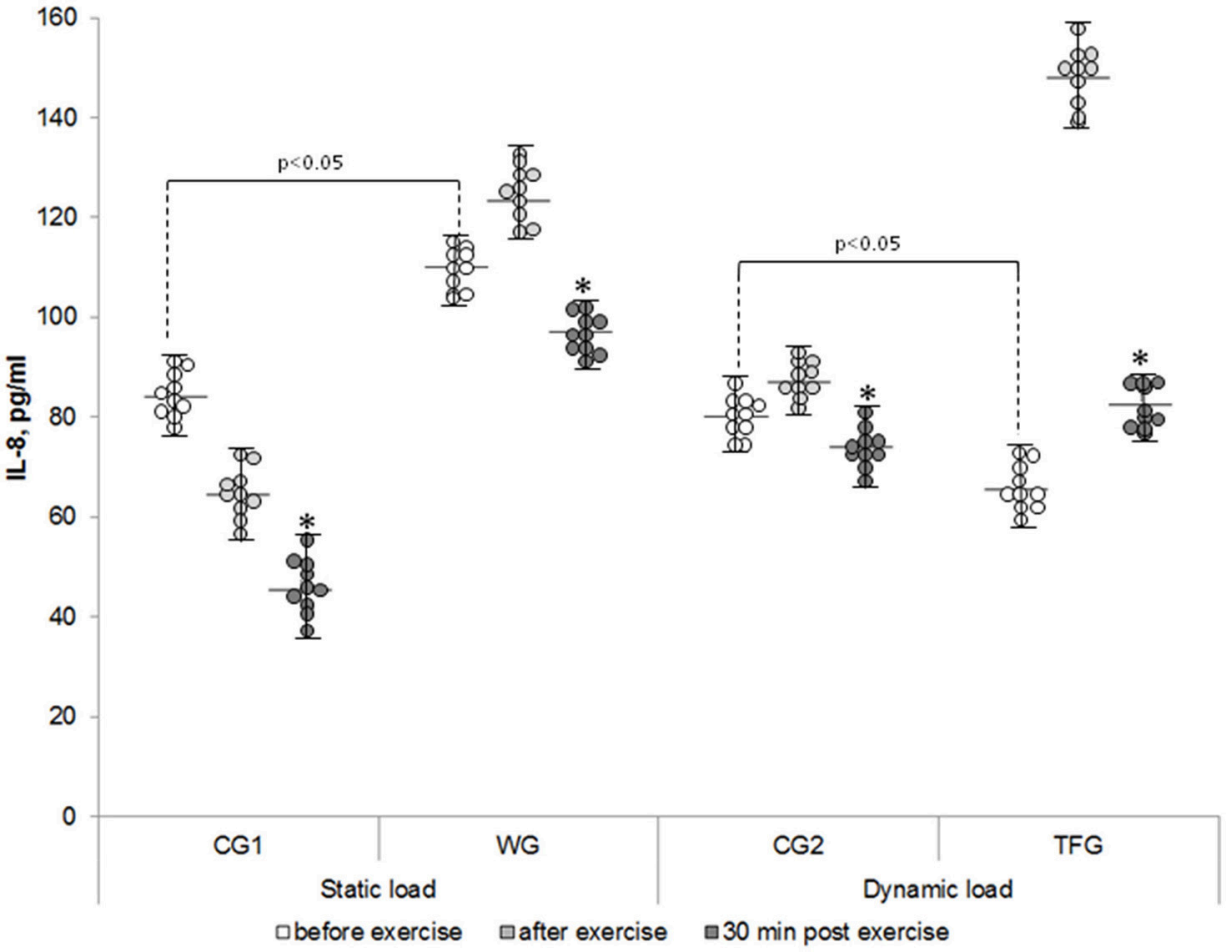
**The individual variability in plasma IL-8 content in the weightlifting (WG), track and field (TFG) athletes and untrained age-matched volunteers**. Horizontal and vertical bars correspond to mean values and standard deviations, respectively. ^*^p < 0.05 compared to the value after exercise.

**Figure 3:**
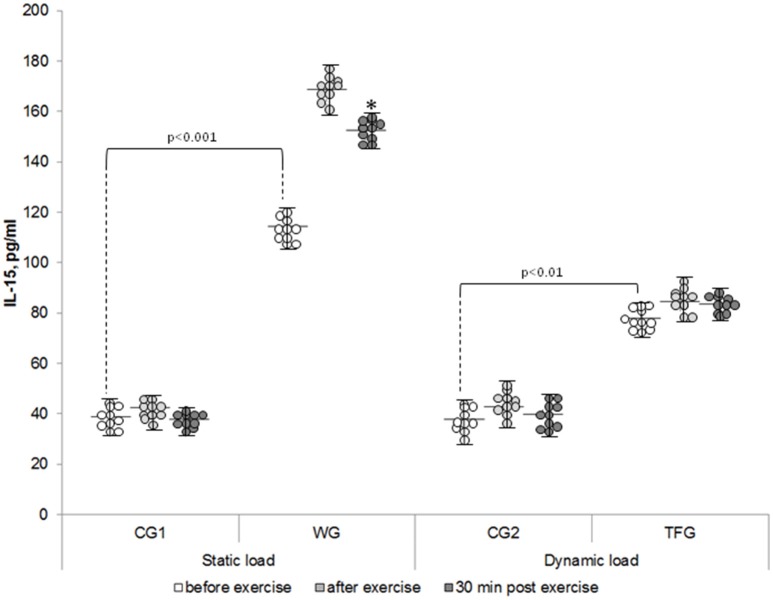
**The individual variability in plasma IL-15 content in the weightlifting (WG), track and field (TFG) athletes and untrained age-matched volunteers**. Horizontal and vertical bars correspond to mean values and standard deviations, respectively. ^*^p < 0.05 compared to the value after exercise.

**Figure 4:**
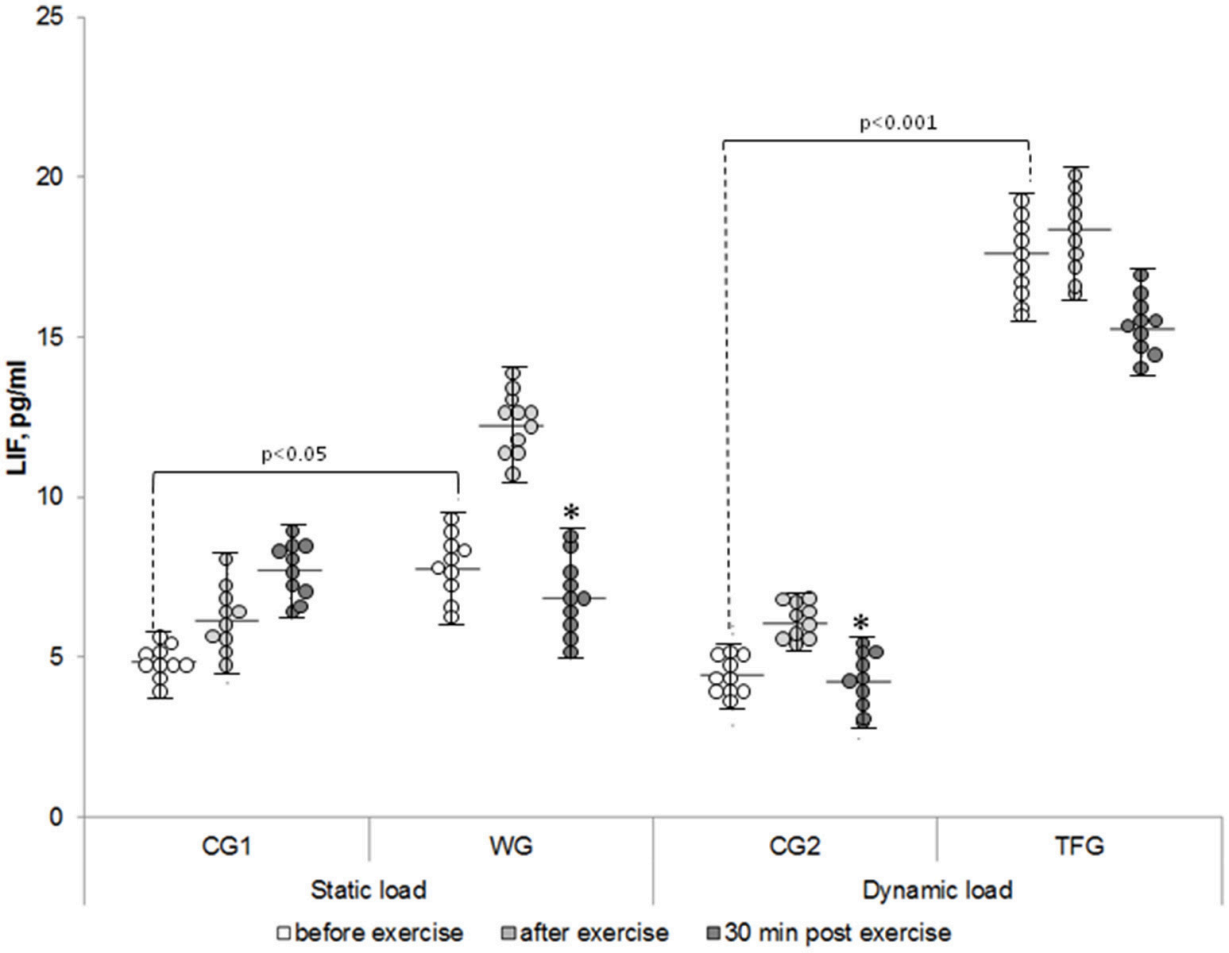
**The individual variability in plasma LIF content in the weightlifting (WG), track and field (TFG) athletes and untrained age-matched volunteers**. Horizontal and vertical bars correspond to mean values and standard deviations, respectively. ^*^p < 0.05 compared to the value after exercise.

The baseline level of IL-8 in the plasma of the weightlifting and track and field athletes was increased by ~30% and decreased by 20%, respectively, as compared to corresponding control groups (*p* < 0.05) (Figure 2). In both the weightlifting and the track and field athletes, the baseline concentration of IL-15 was increased as compared to the untrained volunteers by ~3 (*p* < 0.001)- and 2-fold (*p* < 0.01), respectively (Figure 3). We detected 4–5 pg/ml LIF in the plasma of the untrained volunteers, which was ~2-, 10-, and 20-fold less than that of IL-6, IL-8, and IL-15, respectively (*p* < 0.05). In the track and field and weightlifting athletes, the plasma content was increased by 1.5 (*p* < 0.01)- and 4-fold (*p* < 0.001) compared to the corresponding controls (Figure 4). The individual variability in plasma cytokines are shown in Figures 1–4.

### The effect of static load on cytokine production

**Figure 5:**
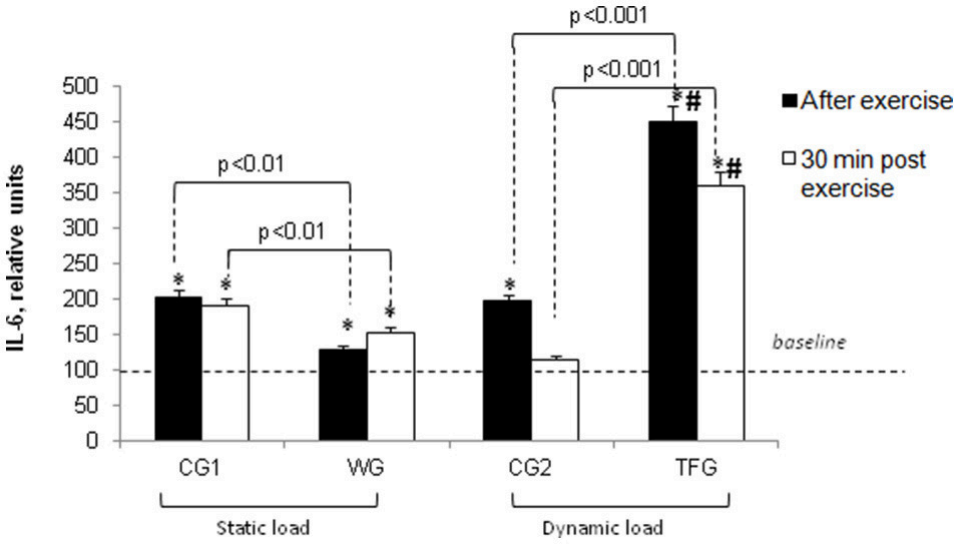
**Effect of static and dynamic exercise on IL-6 content in the plasma of weightlifting (WG) and track and field (TFG) athletes and untrained age-matched volunteers**. In each group, the baseline content of IL-6 was taken as 100%. Means ± S. D. are shown. ^*^Statistical significance level compared to the baseline value. ^#^Statistical significance level compared weightlifting (WG) vs. track and field (TFG) group.

In WG, the *static load* increased plasma IL-6 content by ~25% (*p* < 0.01), which was in contrast to 2-fold elevation seen in both control groups (*p* < 0.01) and 4-fold increment detected in the track and field athletes immediately after the dynamic exercise (*p* < 0.001; Figure 5).

**Figure 6:**
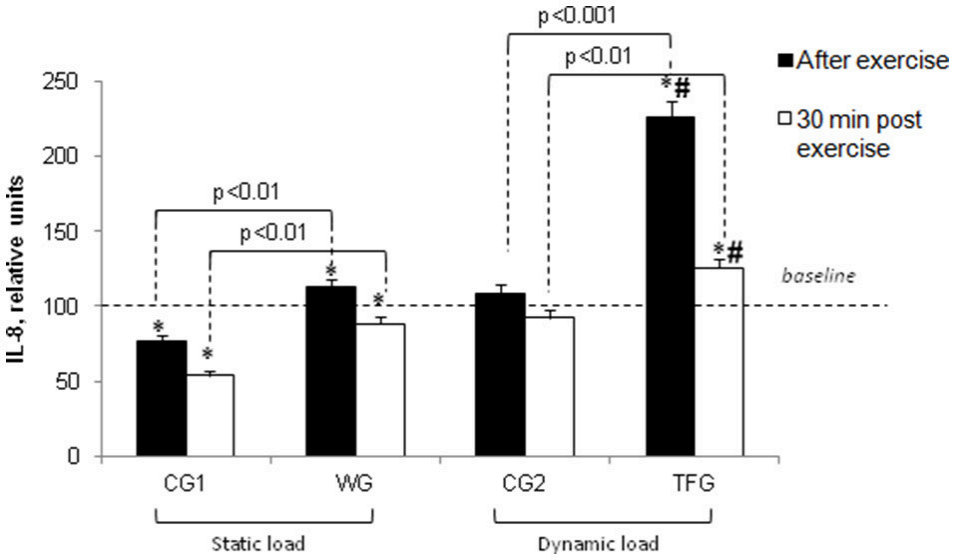
**Effect of static and dynamic exercise on IL-8 content in the plasma of weightlifting (WG) and track and field (TFG) athletes and untrained age-matched volunteers**. In each group, the baseline content of IL-8 was taken as 100%. Means ± S. D. are shown. ^*^Statistical significance level compared to the baseline value. ^#^Statistical significance level compared weightlifting (WG) vs. track and field (TFG) group.

We found a very modest elevation of IL-8 in plasma of athletes subjected to static exercise (from 109.93 ± 1.63 to 123.29 ± 2.92 pg/ml, Figure 2) (*p* < 0.05), whereas in the untrained volunteers the weight-holding procedure decreased this parameter by ~25% (*p* < 0.001, Figure 6).

**Figure 7:**
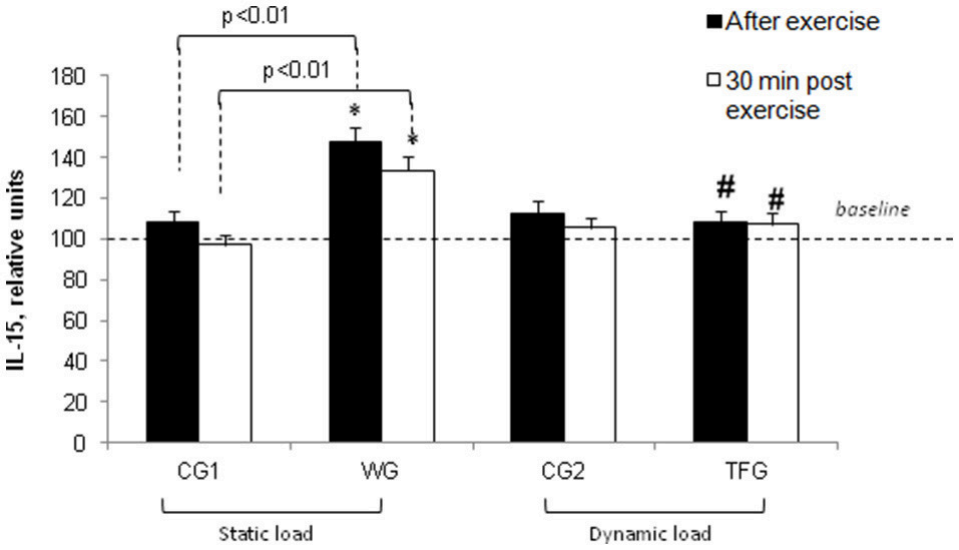
**Effect of static and dynamic exercise on IL-15 content in the plasma of weightlifting (WG) and track and field (TFG) athletes and untrained age-matched volunteers**. In each group, the baseline content of IL-15 was taken as 100%. Means ± S. D. are shown. ^*^Statistical significance level compared to the baseline value. ^#^Statistical significance level compared weightlifting (WG) vs. track and field (TFG) group.

**Figure 8:**
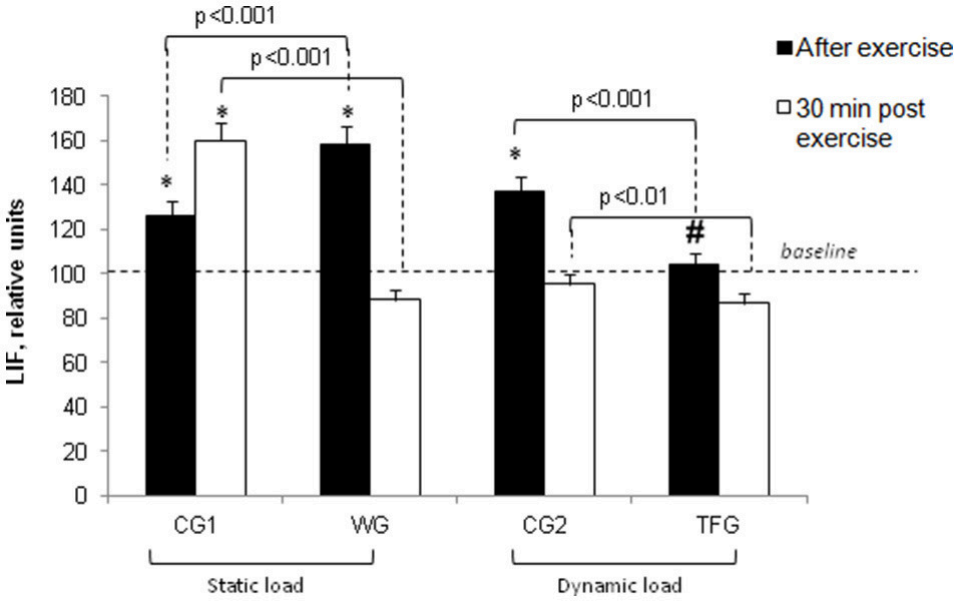
**Effect of static and dynamic exercise on LIF content in the plasma of weightlifting (WG) and track and field (TFG) athletes and untrained age-matched volunteers**. In each group, the baseline content of LIF was taken as 100%. Means ± S. D. are shown. ^*^Statistical significance level compared to the baseline value. ^#^Statistical significance level compared weightlifting (WG) vs. track and field (TFG) group.

For the weightlifting athletes, resistance exercise led to elevation of IL-15 from 114.36 ± 5.92 to 168.55 ± 7.64 pg/ml (*p* < 0.001), but it had no impact on this parameter in the untrained volunteers (38.96 ± 2.52 and 42.31 ± 1.75 pg/ml) (Figures 3, 7). In the athletes and untrained volunteers, the static exercise led to elevation of plasma LIF by ~60 and 30%, respectively (*p* < 0.02, Figure 8).

### The effect of dynamic load on cytokine production

Immediately after completion of dynamic exercise, the plasma content of IL-6 was increased by ~4-5- and 2-fold in the track and field athletes and corresponding controls (CG2), respectively (*p* < 0.001) (Figure 5). It should be noted, however, that absolute values of plasma IL-6 content in TFG remained less compared to WG subjected to static exercise (Figure 1, *p* < 0.01).

In the track and field athletes, dynamic exercise increased plasma IL-8 by ~2-fold (*p* < 0.001) without significant impact in the untrained CG2 volunteers (Figure 6). This increment was 1.9-fold higher compared with negligible impact of static load in the weightlifting athletes (*p* < 0.001, Figure 6).

Endurance training did not affect plasma IL-15 in the track and field athletes as well as in the corresponding control group CG2 (Figures 3, 7). This observation is contrasting with 1.5-fold elevation of plasma IL-15 seen in WG subjected to static load (*p* < 0.01, Figure 7).

Unlike 1-5-fold elevation of LIF in the plasma of the weightlifting athletes observed immediately after the static load (*p* < 0.001; Figure 8), the dynamic exercise had no impact on LIF plasma in the TFG athletes and increased it by about 35% in the untrained CG2 volunteers (*p* < 0.05, Figure 8).

### Plasma cytokine content in 30 min of exercise

Thirty minutes after completing the exercise, IL-6 plasma content remained increased in TFG athletes (*p* < 0.001) but was almost completely normalized in the CG2 volunteers subjected to pedaling on the bicycle ergometer (Figure 5). In 30 min of static load, IL-6 concentration remained elevated in the group of weightlifters and in the control CG1 group as compared with corresponding baseline values (*p* < 0.001) (Figures 1, 5). Thirty minutes after completing the exercise, IL-6 plasma content remained increased by 2-fold in TFG vs. WG (*p* < 0.001, Figure 5).

At the same time interval, IL-8 was decreased by about 2-fold in endurance-trained athletes but remained elevated by 20% compared to its baseline values (*p* < 0.01), whereas in the other groups its content dropped by less than 25% (*p* < 0.05) (Figures 2, 6).

The increment of IL-15 content seen in the weightlifting athletes was preserved 30 min after the mass-holding exercise (*p* < 0.001) and remained unchanged in TFG athletes and CG2 volunteers subjected to pedaling on the bicycle ergometer (Figures 3, 7).

We note that 30 min after static load, the LIF concentration in plasma was increased by ~25% in untrained volunteers (CG1) (*p* < 0.05) but, reached the baseline value in both groups of athletes as well as in untrained volunteers subjected to cyclic exercise (Figures 4, 8). There were no differences of LIF plasma content between the TFG and WG (Figure 8).

## Discussion

The data obtained in the present study led us to two major conclusions. *First*, the impact of static and dynamic exercise on plasma cytokine content is drastically different. Thus, consistent with previous reports (Ostrowski et al., 1999; Steensberg et al., 2000; Fisher, 2006), we observed that endurance exercise sharply increased IL-6 and IL-8 content in the plasma of trained athletes. In contrast to dynamic load, the weightlifting procedure had negligible impact on these parameters in strength exercise-trained athletes (Figures 5, 6). Unlike IL-6 and IL-8, dynamic exercise had no any statistically significant impact on IL-15 and LIF, whereas static load increases the content of these cytokines by ~50% (Figures 7, 8). *Second*, both dynamic and static exercises differentially affect cytokine content in plasma of athletes and untrained persons. Thus, 2-fold increment of IL-8 content seen in athletes subjected to endurance exercise was absent in untrained individuals (Figure 6), whereas the increase in IL-15 triggered by static load in the plasma of weightlifting athletes was not registered in the control group (Figure 7). This finding probably underlies the individual variability of exercise-induced cytokine production seen in the majority of investigations (for review, see Pedersen and Febbraio, 2008; Peake et al., 2015).

The distinct effect of dynamic and static exercise on cytokine production in athletes and untrained volunteers might be explained by the impact of different cell types as well as by different mechanisms of excitation–transcription coupling. Indeed, side-by-side with myocytes, skeletal muscle contains fibroblasts, pericytes, adipocytes, and motor neurons whose contributions to overall myokine production are poorly defined (Peake et al., 2015). The relative content of these cells as well as their impact in exercise-induced cytokine release might be different in trained and untrained adults. In addition to heterogeneity of cell types, it was shown that skeletal muscle myocytes can be subdivided into three different phenotypes, and their relative content is differentially affected by aerobic and resistant training interventions (Fitts and Widrick, 1996; Egan and Zierath, 2013). Using the Affymetrix technology, it was shown that transcriptomic changes triggered by resistance exercise are most pronounced in fast-twitch muscle IIa type fibers (Raue et al., 2012).

Hypoxia-inducible factor (HIF-1α) and AMP- and ${\text{Ca}}_{\text{i}}^{2+}$-sensitive protein kinases and phosphoprotein phosphatases as well as novel [Na^+^]_i_/[K^+^]_i_-mediated signaling are involved in excitation–transcription coupling in skeletal muscle (Gundersen, 2011; Kapilevich et al., 2015). HIF-1α is translocated to the nucleus, where it forms HIF-1α/HIF-1β complex and trigger transcription of dozens of genes including vascular endothelial growth factor (VEGF) and endothelial nitric oxide synthase (eNOS) (Ke and Costa, 2006). Importantly, unlike dynamic load, static exercise leads to blood vessel occlusion and local hypoxia and is accompanied by accumulation of VEGF and eNOS mRNA and protein in rat skeletal muscle (Rodriguez-Miguelez et al., 2015). However, the role of this pathway in exercise-induced cytokine release has not yet been explored. The role of AMPK in exercise-induced myokine expression is supported by data showing that exercise-induced IL-15 production was decreased in mice lacking both β1 and β2 AMPK subunits in skeletal muscle (Crane et al., 2015). Coffey and coworkers demonstrated that AMPK phosphorylation increased in muscle biopsies after cycling in strength-trained but not endurance-trained subjects (Coffey et al., 2006). It should be noted, however, that contraction-mediated IL-6 expression was normal in muscle-specific AMPK α2 knockout mice (Lauritzen et al., 2013).

Besides triggering muscle contraction, elevation of [Ca^2+^]_i_ from ~0.1 to 1 μM affects the expression of hundreds of genes, i.e., the phenomenon termed excitation–transcription coupling (Santana, 2008; Gundersen, 2011; Ma et al., 2011). Indeed, treatment of rat soleus muscle with the Ca^2+^ ionophore ionomycin for 1 h resulted in 5-fold elevation of IL-6 mRNA content (Holmes et al., 2004). Later, Whitham and coworkers found that exposure of C_2_C_12_ myotubes to the less selective Ca^2+^ ionophore A23187 sharply increased IL-6 transcription (Whitham et al., 2012). Using the same *in vitro* exercise model, it was shown that the extracellular Ca^2+^ chelator EGTA diminishes by 2-fold the EPS-induced accumulation of CXL (Nedachi et al., 2009).

Sustained excitation of skeletal muscle results in dissipation of the transmembrane gradient of monovalent cations due to Na^+^ influx and K^+^ efflux via voltage-gated and Ca^2+^-sensitive ion channels. Thus, both in humans and in experimental animals long-term exercise increased [Na^+^]_i_ in skeletal muscles by 3-4-fold and decreased [K^+^]_i_ to 50%, which was accompanied by sharp elevation of [K^+^] in plasma and interstitial fluid (Sejersted and Sjøgaard, 2000; McDonough et al., 2002; Cairns and Lindinger, 2008; McKenna et al., 2008; Murphy et al., 2008). These findings suggest that elevation of the [Na^+^]_i_/[K^+^]_i_ ratio *per se* is sufficient to trigger myokine production (Kapilevich et al., 2015). Indeed, in several cell types, sustained elevation of the [Na^+^]_i_/[K^+^]_i_ ratio resulted in augmented expression of several myokines, including IL-6 (Koltsova et al., 2012). To examine the relative contribution of ${\text{Ca}}_{\text{i}}^{2+}$-mediated and ${\text{Ca}}_{\text{i}}^{2+}$-independent signaling, we compared transcriptomic changes triggered by elevation of the [Na^+^]_i_/[K^+^]_i_ ratio in control and Ca^2+^-depleted cells. Surprisingly, Ca^2+^ depletion increased rather than decreased the number of ubiquitous and cell-type specific [Na^+^]_i_/[K^+^]_i_-sensitive genes (Koltsova et al., 2012). Among the ubiquitous [Na^+^]_i_/[K^+^]_i_-sensitive genes upregulated independently of the presence of Ca^2+^ chelators, we found the canonical myokine IL-6. Recently, we reported that extracellular Ca^2+^ chelators sharply increase permeability of the plasma membrane for monovalent ions, resulting in elevation of the [Na^+^]_i_/[K^+^]_i_ ratio (Koltsova et al., 2015). We also demonstrated that in vascular smooth muscle cells hypoxia-induced transcriptomic changes are at least partially triggered by HIF-1α-independent, [Na^+^]_i_/[K^+^]_i_-mediated, excitation–transcription coupling. (Koltsova et al., 2014).

It is unclear, however, whether or not HIF-1α-, [Ca^2+^]_i_-, and [Na^+^]_i_/[K^+^]_i_-dependent transcription-translation-cytokine release pathways could operate quickly enough to induce increased plasma cytokine levels immediately after such a short exercise bout. Thus, additional experiments should be performed to clarify their relative role in the distinct impact of dynamic and static exercises on cytokine accumulation in the plasma of athletes and untrained volunteers.

## Conclusion

Our results show the distinct impact of static and dynamic exercise on plasma cytokine content. They also demonstrate that both types of exercises differentially affect cytokine content in plasma of athletes and untrained persons. The impact of different cell types and mechanisms of excitation–transcription coupling in the distinct effect of dynamic and static exercise on cytokine production in athletes and untrained volunteers should be explored in forthcoming studies.

## Ethics statement

This study was carried out in accordance with the recommendations of “Commission on Bioethics of Biology Faculty of the National Research Tomsk State University” with written informed consent from all subjects. All subjects gave written informed consent in accordance with the Declaration of Helsinki. The protocol was approved by the “Commission on Bioethics of Biology Faculty of the National Research Tomsk State University.”

## Author contributions

LK, the development of the experiment plan, writing of the article; AZ, carrying out the experiment, analysis of the experimental data, writing of the article; AK, curetting of the experiment, writing of the article; TK, carrying out the experiment, the analysis of the experimental data; SO, management of project, writing of the article, correction and approval of the final version of the text.

## Funding

This study was supported by a grant from the Russian Science Foundation (16-15-10026).

### Conflict of interest statement

The authors declare that the research was conducted in the absence of any commercial or financial relationships that could be construed as a potential conflict of interest.
